# A Diels-Alder polymer platform for thermally enhanced drug release toward efficient local cancer chemotherapy

**DOI:** 10.1080/14686996.2021.1939152

**Published:** 2021-06-24

**Authors:** Nanami Fujisawa, Masato Takanohashi, Lili Chen, Koichiro Uto, Yoshitaka Matsumoto, Masayuki Takeuchi, Mitsuhiro Ebara

**Affiliations:** a Research Center for Functional Materials, National Institute for Materials Science (NIMS), Tsukuba, Japan; b Graduate School of Pure and Applied Sciences, University of Tsukuba, Tsukuba, Japan; c Department of Radiation Oncology, University of Tsukuba Hospital, Tsukuba, Japan; d Graduate School of Advanced Engineering, Department of Materials Science and Technology, Tokyo University of Science, Katsushika-ku, Japan

**Keywords:** Diels-alder reaction, cancer, chemotherapy, gemcitabine, alternating magnetic field, local drug delivery, 30 Bio-inspired and biomedical materials; 211 Scaffold/Tissue engineering/Drug delivery

## Abstract

We reports a novel thermally enhanced drug release system synthesized via a dynamic Diels-Alder (DA) reaction to develop chemotherapy for pancreatic cancer. The anticancer prodrug was designed by tethering gemcitabine (GEM) to poly(furfuryl methacrylate) (PFMA) via *N*-(3-maleimidopropionyloxy)succinimide as a linker by DA reaction (PFMA-L-GEM). The conversion rate of the DA reaction was found to be approximately 60% at room temperature for 120 h. The reversible deconstruction of the DA covalent bond in retro Diels-Alder (rDA) reaction was confirmed by proton nuclear magnetic resonance, and the reaction was significantly accelerated at 90 °C. A PFMA-LGEM film containing magnetic nanoparticles (MNPs) was prepared for thermally enhanced release of the drug via the rDA reaction. Drug release was initiated by heating MNPs by alternating magnetic field. This enables local heating within the film above the rDA reaction temperature while maintaining a constant surrounding medium temperature. The MNPs/PFMA-L-GEM film decreased the viability of pancreatic cancer cells by 49% over 24 h. Our results suggest that DA/rDA-based thermally enhanced drug release systems can serve as a local drug release platform and deliver the target drug within locally heated tissue, thereby improving the therapeutic efficiency and overcoming the side effects of conventional drugs used to treat pancreatic cancer.

## Graphical abstract

**Figure:**
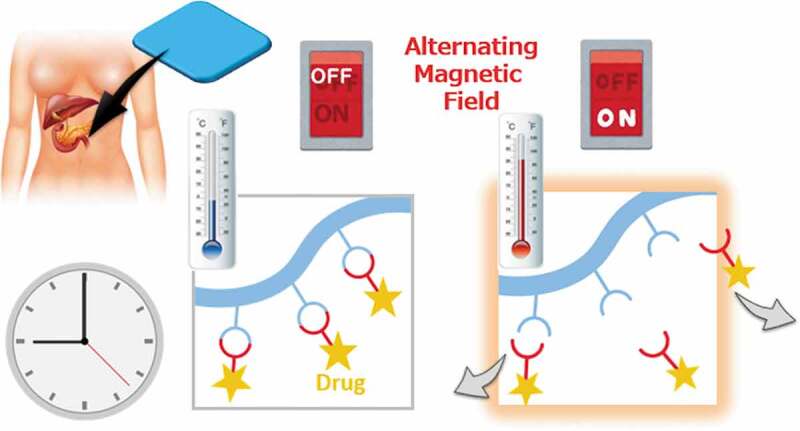

## 1. Introduction

Cancer is the leading cause of death in the developed world, as one in three individuals develops cancer during their lifetime. Particularly, pancreatic cancer is lethal, and approximately 95% of patients with pancreatic cancer die as they experience few or no symptoms during the early stages [1]. While systemic drug delivery is commonly used for cancer chemotherapy, it is often difficult to deliver the drug to the target location because of limited extravasation from the bloodstream into the target tissue. Over time, there is often a need for higher drug dosages to maintain the requisite local concentration during the treatment period [2–5]. However, higher dosages increase the risk of toxicity and the occurrence of adverse side effects. The situation becomes even more challenging when the blood supply is minimal or preliminarily destroyed due to trauma or surgery. To overcome this major limitation, it is desirable to deliver active ingredients locally, targeting the medication directly to the disease site [6]. This can be done by implanting a drug reservoir directly into the target area and releasing the drug over the desired period and rate; this is phenomenon is called ‘local drug delivery’. Injectable hydrogels have been the most extensively researched materials for use as carriers of therapeutic agents [7,8]. One of the important advantages of injectable hydrogels is their low invasiveness and high usability. Despite these advantages, the development of injectable hydrogels may face some challenges in meeting various clinical requirements.

We have been developing implantable nanofiber or film-based platforms for localized drug delivery [9–12]. This system is essentially aimed at delivering and retaining sufficient quantities of active drug molecules within an adequate period. For example, we have demonstrated the enhanced treatments of lung cancer, skin cancer, prostate cancer, and liver cancer using electrospun nanofiber meshes incorporating paclitaxel [13], imiquimod [8,14], Hemagglutinating Virus of Japan Envelope (HVJ-E) [15], and micro RNA 145 [16], respectively. Furthermore, thermo-responsive nanofiber meshes have been used in conjunction with magnetic nanoparticles (MNPs) for cancer chemotherapy/thermotherapy [17,18]. The combination of cancer drugs and hyperthermia was shown to have a greater impact on cell apoptosis, with the advantage of controlled release using an alternating magnetic field (AMF). Recently, the effectiveness of hyperthermia has been further enhanced through the inhibition of heat shock protein activity by releasing the inhibitor 17-allylamino-17-demethoxygeldanamycin from the mesh [19]. Although these systems are highly desired, ON/OFF drug release is not controlled and remains a challenge. Therefore, the risk of toxicity remains unclear.

**Figure 1.**
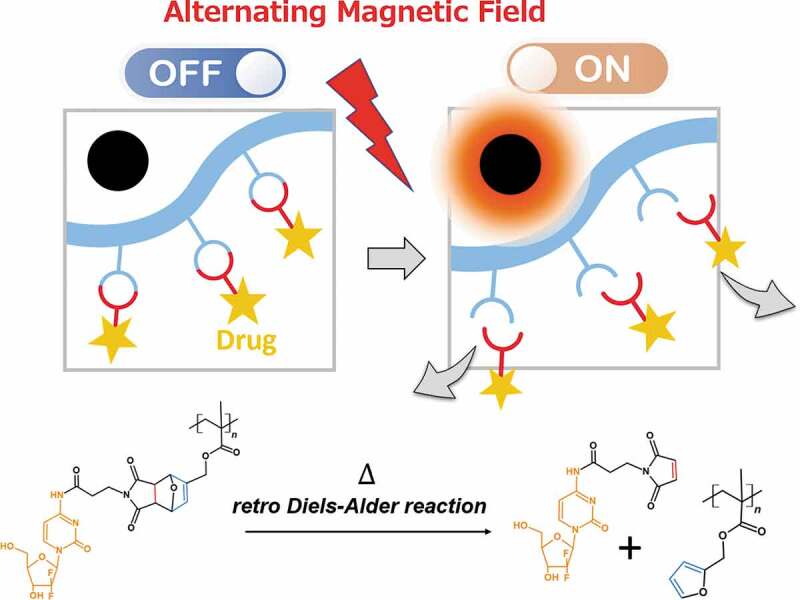
Schematic of drug release from polymer sidechain by retro Diels-Alder reaction. The polymer conjugates gemcitabine (GEM) via thermo-responsive covalent bonding. Magnetic nanoparticles generate heat near the drug release system. The mesh releases the drug when activated by alternating magnetic field

To improve thermally enhanced drug release, we focused on dynamic and reversible covalent chemistry, such as the Diels-Alder (DA) reaction. The DA reaction is a chemically selective [4 + 2] cyclization between a diene and a dienophile, electron-donating, and electron-withdrawing groups, respectively, which increases its reactivity. This covalent bond can also reversibly return to its original form upon heating. This is called retro Diels-Alder (rDA) reaction. One of the benefits of the DA reaction in the biomedical field is that it can be used in an aqueous medium [20]. Oluwasanmi et al. employed a DA reaction linker to conjugate gemcitabine onto the surface of hybrid nanoparticles and demonstrated drug release by laser irradiation via surface plasmon resonance [21]. Hammad et al. developed doxorubicin-conjugated magnetic nanoparticles via a DA linker [22]. The drug was released through the rDA reaction upon exposure to alternating magnetic fields. In the aforementioned studies, drugs were directly conjugated to the surface of metal particles. Limitations in terms of their fabrication variations, such as films, fibers, and hydrogels, were noted in those studies. To overcome these limitations, in this study, anticancer drug gemcitabine (GEM) was directly conjugated to poly(furfuryl methacrylate) (PFMA) via *N*-(3-maleimidopropionyloxy)succinimide as a linker by the DA reaction (PFMA-L-GEM) (Figure 1). The conversion rate of DA/rDA reaction was determined using proton nuclear magnetic resonance (^1^H NMR) spectroscopy. A magnetic nanoparticle-incorporated PFMA-L-GEM film was prepared. Further, the release of the drug was initiated by heat generation of MNPs after AMF irradiation. *In vitro* experiments demonstrated the cytotoxicity of released L-GEM on pancreatic cancer cells.

## 2. Experimental details

### 2.1. Materials

Furfuryl methacrylate (FMA), heat-inactivated fetal bovine serum (FBS), 1% L-glutamine, 1% penicillin/streptomycin, 0.25% trypsin-EDTA, and RPMI-1640 medium were purchased from Sigma-Aldrich Japan (Tokyo, Japan). *N*-Succinimidyl-3-maleimidopropionate (linker), 1, 1, 1, 3, 3, 3-hexafluoro-2-propanol (HFIP), and gemcitabine (GEM) were purchased from Tokyo Chemical Industry Co., Ltd. (Tokyo, Japan). 2ʹ2-Azobis (isobutyronitrile) (AIBN), chloroform-*d* (containing 0.1% tetramethylsilane (TMS)), dimethyl sulfoxide-*d*_6_ (containing 0.05% v/v TMS), dimethylformamide (DMF), dichloromethane, 1, 1, 2, 2-tetrachloroethane-*d*_2_, and triethylamine were purchased from FUJIFILM Wako Pure Chemical Corporation (Osaka, Japan). Iron (III) oxide nanoparticles (particle size <50 nm) were purchased from Ferrotec Material Technologies Corporation (Tokyo, Japan). MIAPaCa-II cells were purchased from RIKEN Bioresource center (Tsukuba, Japan). AlamarBlue® reagent was purchased from Thermo Fisher Scientific (Massachusetts, USA). Phosphate-buffered saline (PBS) was purchased from Nakalai Tesuque (Kyoto, Japan) (pH 7.4, 0.1% w/v).

### 2.2. Synthesis of PFMA

As shown in Scheme S1 (Supplementary Information), the polymerization of FMA was carried out via free radical polymerization. As conventional radical polymerization can easily lead to excessive gel formation, the concentration was carefully adjusted. Briefly, FMA and AIBN (0.01 mol% of total monomer concentration) were dissolved in 20 mL of DMF. The total monomer was 50 mmol. The polymerization was carried out at 60°C for 20 h, after nitrogen was bubbled. After polymerization, AIBN, unreacted monomers, impurities, and solvent were removed by dialysis against DMF and distilled with dichloromethane for 3 days. The dialyzed solutions were then evaporated. The chemical structure of the obtained polymer was confirmed via ^1^H NMR spectroscopy at 400 MHz (JEOL, Tokyo, Japan). All NMR samples were prepared in deuterated solvents, with all values quoted in ppm relative to TMS as an internal reference. The average molecular weight (*M*_n_) and polydispersity index (PDI) of the homopolymers were determined via gel permeation chromatography (GPC, JASCO International, Tokyo, Japan) using DMF with lithium bromide (LiBr, 10 mM) (Tosoh Corporation, Tokyo, Japan) as the eluent.

### 2.3 Conjugation of maleimide-linker to PFMA (DA reaction)

*N*-Succinimidyl-3-maleimidopropionate (linker) (0.80 g) was dissolved in dichloromethane (20 mL), followed by PFMA (0.50 g). The mixture was vigorously stirred, which resulted in the formation of a slurry. The slurry was then stirred for 120 h at room temperature. A white solid (PFMA-L) was collected by evaporation. The chemical structure was confirmed by ^1^H NMR spectroscopy using chloroform-*d* as the solvent.

### 2.4. Deconstruction of rDA reaction

PFMA-L (2.0 mg) and 0.7 mL of 1, 1, 2, 2-tetrachloroethane-*d*_2_ were added to an NMR tube. ^1^H NMR measurements were conducted under different temperatures (25, 37, 45, and 90 °C) at different times (~60 min). Thermal analysis of PFMA-L was also conducted using differential scanning calorimetry (DSC) (6100, SEIKO Instruments, Chiba, Japan).

### 2.5. Conjugation of GEM to PFMA-L

GEM was conjugated to PFMA-L by nucleophilic acyl substitution between the activated ester group of PFMA-L and the amine group of gemcitabine. One gram of PFMA-L and 0.6 g of GEM were placed in a round-bottomed flask with 150 mL of HFIP under N_2_ gas. Then, 2 mL of triethylamine was added to the reaction solution. The mixture was allowed to react under stirring at room temperature for 24 h. The product was completely dried under vacuum at room temperature. The structures were characterized by ^1^H NMR spectroscopy with DMSO-*d*_6_ and attenuated total reflection Fourier transform infrared spectroscopy (ATR-FTIR, Thermo Fisher Scientific K.K., Tokyo, Japan).

### 2.6. Preparation of MNP/PFMA-L-GEM films

PFMA-L-GEM (1.13 g) and MNPs (0.90 g) were dissolved in HFIP at 41.5 wt%. The mixture solution was coated on 15 mm glass coverslips at 3000 rpm for 60 s by spin coating (Active Spin Coater, Axel, Osaka, Japan). The MNP/PFMA-L-GEM films were cut into 100 mg film pieces (37.7 mg L-GEM/100 mg film).

### 2.7. Heating potential of MNP/PFMA-L-GEM films

The heating profiles of the MNP/PFMA-L-GEM films were investigated. The film was immersed in 50 mL PBS at 20 °C. The sample was placed in the center of a copper coil and exposed to an AMF (166 kHz and 192 A, HOSHOT2, Alonics Co., Ltd., Tokyo, Japan). The heating profiles were obtained by taking photos using a forward-looking infrared camera (CPA-E6, FLIR Systems Japan K.K., Tokyo, Japan).

### 2.8. In vitro drug release

Drug release studies of the PFMA-L-GEM films were conducted using a dialysis membrane. Briefly, 1 mL of PBS with PFMA-L-GEM film (0.1 g) was loaded into a Spectra/Por® dialysis tubing with a molecular weight cut-off of 10 kDa (Repligen, Massachusetts, USA). The dialysis membrane was immersed in 100 mL PBS. The solutions were stirred, and the rate of drug release was measured at various temperatures (25°C, 37°C, 45°C, and 90°C). Ten milliliters of released L-GEM in PBS were collected, and 10 mL of fresh PBS was added to each sample. The released amount of L-GEM was quantified using a UV-vis spectrophotometer (V-650 spectrophotometer, Jasco, Tokyo, Japan) from a calibration curve (R^2^ = 0.996). Thermal analysis of PFMA-L-GEM was also conducted using DSC as a same way of PFMA-L.

### 2.9. In vitro cytotoxic assay

All *in vitro* experiments in this study were carried out using the pancreatic cancer cell line MIAPaCa-II. MIAPaCa-II cells were grown in RPMI1640 supplemented with 10% FBS, 1% L-glutamine, and 1% penicillin/streptomycin. The cells were maintained at 37 °C in a humidified atmosphere of 5% CO_2_. Subculturing was performed every 2–3 days with 0.25% trypsin-EDTA until the cells were ready for use.

Cell cultures were produced in 96-well plates by seeding 4.0 × 10^3^ MIAPaCa-II cells in their exponential growth phase and incubating them overnight at 37°C in a 5% CO_2_ atmosphere in RPMI-1640 media supplemented with 1% streptomycin/penicillin and 5% fetal bovine serum. The released L-GEM from the PFMA-L-GEM films under different conditions was collected and added to each cell culture well. Cytotoxicity was confirmed via the AlamarBlue® assay after incubation at 37°C for 24 h.

## 3. Results and discussion

### 3.1. DA reaction

**Scheme 1.**
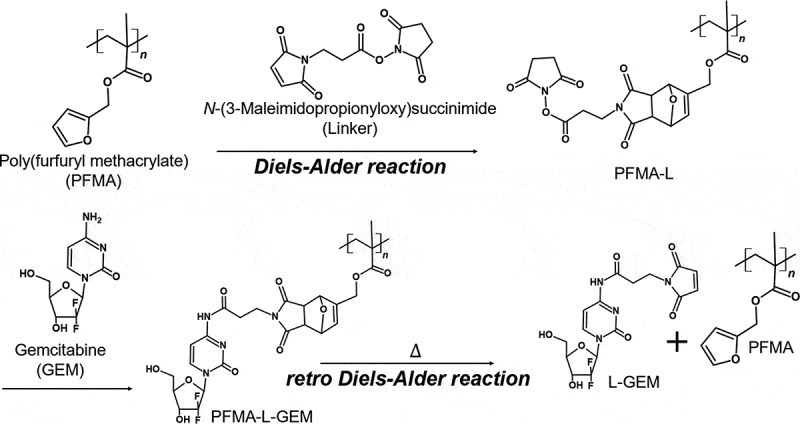
Reaction scheme for the synthesis of PFMA-L-GEM by the Diels-Alder cycloadduct formation and L-GEM release by retro Diels-Alder reaction by heating

**Figure 2.**
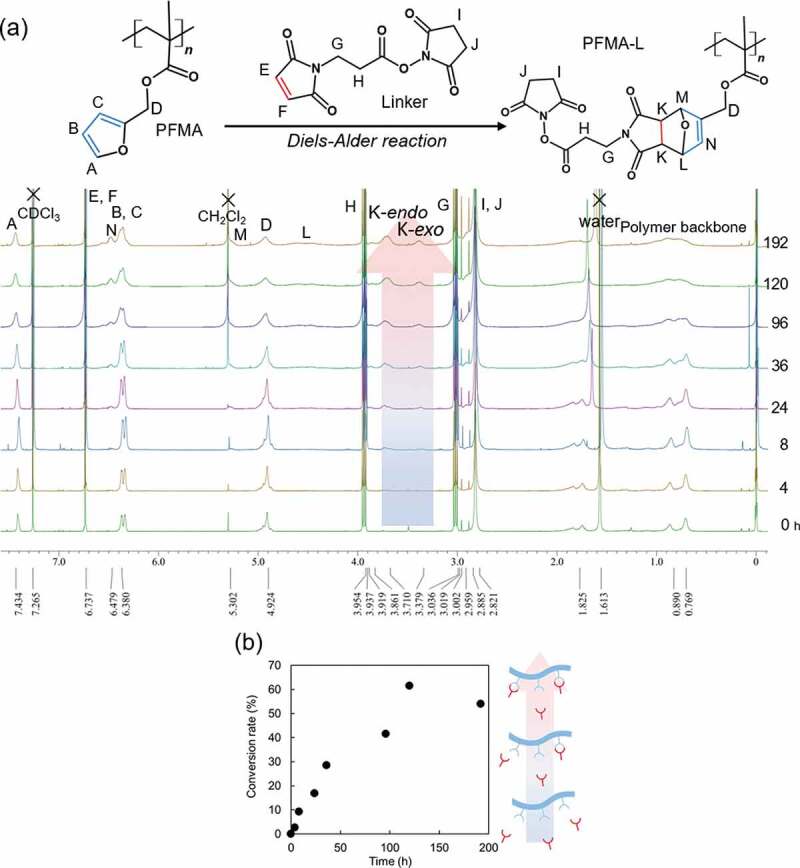
(a) ^1^H NMR spectra of Diels-Alder reaction between PFMA and linker. (b) Conversion rate of Diels-Alder reaction between PFMA and Linker

PFMA was prepared by free radical polymerization with a yield exceeding 62% (Scheme S1 and Figure S1 in Supplementary Information). The molecular weight (Mn) was estimated to be 72,000 g mol-1 with a PDI of 1.30 (Figure S2 in Supplementary Information). Immobilization of the thermally labile linker onto the PFMA molecule was achieved by the DA reaction (Scheme 1). PFMA-L is insoluble in water due to its high hydrophobicity. Dichloromethane was used as the reaction solvent for the DA reaction. The progress of the DA reaction was confirmed via ATR-FTIR and ^1^H NMR spectroscopy with chloroform-*d* (Figure 2(a)). The ATR-FTIR spectra revealed that pronounced peaks at 1013 cm^−1^ were observed for PFMA, which is presumed to be the furan ring. This peak disappears after the reaction with linker (Figure S3 in Supplementary Information) [23]. The peaks of PFMA-L were observed at 6.47 (N), 5.30 (M), and 4.52 (L) ppm. Free furan and free maleimide groups were assigned at 7.43 (A), 6.38 (B, C), and 6.73 (E, F) ppm, respectively. The conversion rate was calculated based on the integral ratio of resonance at 3.70 (K-endo) and 3.38 (K-exo) ppm. The conversion rate of the DA reaction between the PFMA and linker (Figure 2(b)) increased with time and saturated at approximately 60% at 120 h. The reaction speed of the DA reaction is highly dependent on the reaction environment, such as the reaction solvent or temperature. For example, the DA reaction time in water was reported to be 30 min at 60 °C [24], whereas the DA reaction time in diethyl ether was 7 days at room temperature under a N_2_ atmosphere [21]. In addition, it has been difficult to assign every peak observed for the polymeric materials because of the broad NMR signals obtained when compared to the signals obtained for low molecular weight compounds [25,26]. Therefore, careful observation of the conversion rate using ^1^H NMR measurement is important (Figure S4 in Supplementary Information).

### 3.2. Retro DA reaction

**Figure 3.**
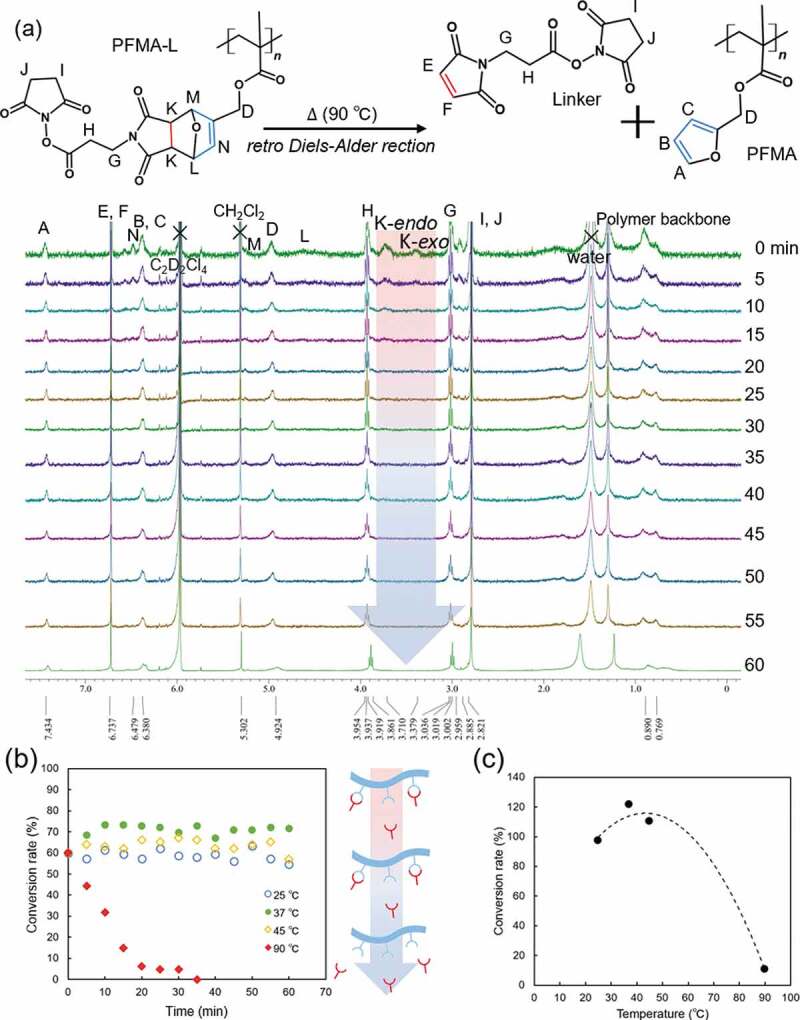
(a) ^1^H NMR spectra of PFMA-L of retro Diels-Alder reaction at 90 °C (b) Conversion rate of retro Dies-Alder reaction between PFMA and Linker (c) conversion rate plots vs. reaction temperature at 20 min

For the rDA reaction, 1, 1, 2, 2-tetrachlorethane-*d*_2_ solution was used because it does not dissolve in aqueous media. Another reason for the use of tetrachloroethane is its high boiling temperature; therefore, the rDA reaction can be performed at higher temperatures. The conversion rate was calculated from the integrated area of the peak using ^1^H NMR spectroscopy in the same manner as for the DA reaction. Figure 3(a) shows the time-dependent changes in the ^1^H NMR peaks for the rDA reaction at 90 °C. Figure 3(b) shows a comparison of the time-dependent conversion rates at different temperatures. The vertical axis indicates the conversion rate of the rDA reaction. According to this result, 100% of the rDA reaction was observed at 90 °C for approximately 30 min of heating. In contrast, no changes in the conversion rate were observed below 45 °C. Interestingly, the conversion rate exceeded 100% when the reaction was conducted at 37 and 45 °C. These results indicate that the DA reaction proceeded in opposite directions at these temperatures in tetrachloroethane. Other researchers have also reported that the threshold temperature of thermal breakdown of the produced ring is unstable depending on the reaction environment, especially the temperature or the reaction solvent [24,27–29]. Froidevaux et al. reported that there are three distinct regions for the DA/rDA reaction, and the reaction does not increase or decrease linearly [30]. In addition, the DA reaction usually leads to a mixture of two diastereomers – endo and exo. In our material, the endothermic peaks for endo and exo products appear at approximately 50–110°C and 130–142°C in the dry state, respectively in the DSC measurements (Figure S5 in Supplementary Information). In addition, the endothermic reaction due to the rDA reaction was not observed like the first cycle in the second heating cycle (Figure S5a, S4b in Supplementary Information). It is considered that the DA reaction was not confirmed in cooling cycle (Figure S5c, S5d in Supplementary Information) because it takes a long time as shown in Figure 2(b). Owing to the effects of the solvents, the observed temperatures were not consistent with those obtained in Figure 3. The heating rate was also considered to affect the reaction kinetics. Therefore, we chose the temperature at 90 °C as the rDA reaction temperature in the present study for the following experiments.

### 3.3. Heat generation

**Figure 4.**
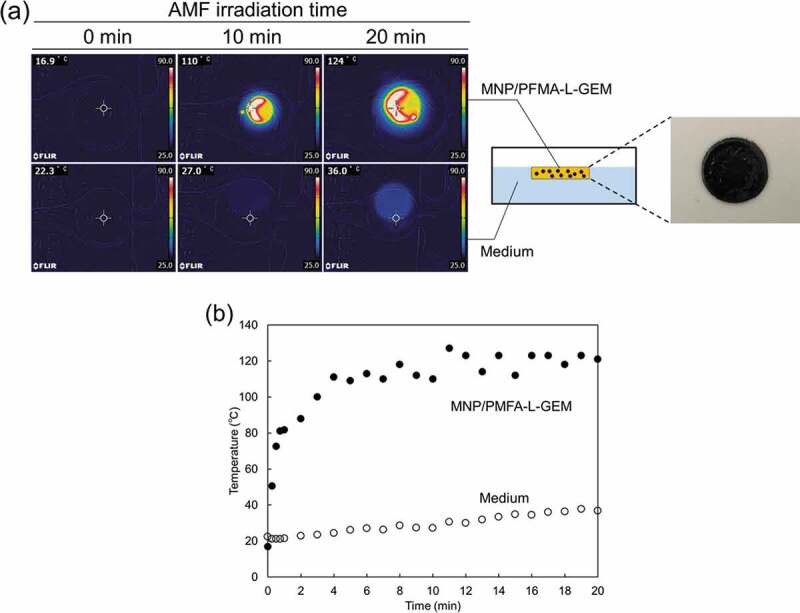
(a) Infrared thermal images and pictural image of PFMA-L-GEM film incorporating MNPs. (b) Heating profile of AMF irradiation to the PFMA-L-GEM film

To examine the thermally enhanced drug release potential of PFMA-L-GEM, MNP-incorporated films were prepared. Iron-oxide MNPs are known to generate large amounts of thermal energy under the influence of AMF of optimal frequency and amplitude. In this experiment, we used AMF at 166 kHz and 192 A, which corresponds to 2.73 × 10^9^ Am^−1^ s^−1^ (specific absorption rate, SAR = 2.68 W g^−1^). The amplitude of AMF was 1.65 × 10^4^ A m^−1^. This intensity of the amplitude of AMF is relevant for clinical use. The MNPs used in this study were Fe_2_O_3_ nanoparticles with diameters <50 nm. The saturated magnetization obtained by vibrating sample magnetometer was 57 emu g^−1^ [17]. Figure 4(a) shows the infrared thermal images of PFMA-L-GEM films loaded with 0.9 g of MNPs. The temperature of the AMF-exposed film rose to above 100°C within 10 min. Figure 4(b) depicts the time-dependent temperature changes of the films during AMF application. The samples showed a sharp increase in temperature during the first 5 min of AMF application before reaching a plateau. We also measured the solution temperature of the surrounding medium, and small changes in temperature increase were observed. These results demonstrated that even if the solution temperature did not increase significantly, the actual local temperature of the MNPs increased above the rDA reaction temperature (> 90°C) to release the drug.

### 3.4. Thermally enhanced drug release

**Figure 5.**
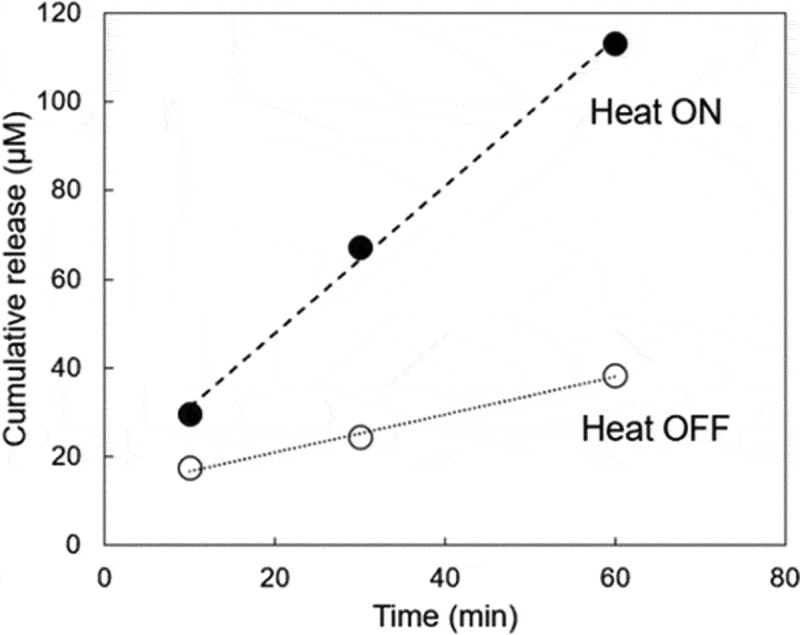
(a) Cumulative drug release from pre-heated (closed circles) and unheated PFMA-L-GEM films (open circles, n = 3)

Figure 5(a) plots the release profile of drug at 37 °C from the PFMA-L-GEM films. The ATR-FTIR spectra revealed that pronounced peaks at 1537 cm^−1^ and 1640 cm^−1^ were observed for L-GEM compared with free GEM, which are presumed to be the amido bond between linker and GEM (Figure S3 in the Supplementary Information). The PFMA-L-GEM film slowly released L-GEM. Approximately 40 μM of L-GEM content was released at 37 °C after 60 min detected via UV-vis measurement at 265 nm (Figure S6 in the Supplementary Information). On the contrary, when heating was conducted, over 100 μM of L-GEM was detected in the solution as released drug, indicating that approximately 13.5 mg of L-GEM from the film was released. The higher release rate observed at elevated temperature corresponds to an increased reverse rate in the rDA reaction. As shown in Figure 4, the local temperature was found to be above 90 °C. Therefore, the accelerated drug release observed is an expected result. A significant observation was that continuous drug release (zero-order) was noted without a significant initial burst release (Figure 4(b)) . This result indicates that the drug release mechanism is based not only on simple diffusion from the film, but also reversible DA/rDA reaction because drug release becomes anomalous or non-Fickian when drug-matrix interaction is much greater than the diffusion time [31]. Therefore, the accelerated release of L-GEM from the films was most likely mediated by the rDA reaction induced by the thermal effect of the MNPs in the film, although complete on-off drug release control was not achieved.

### 3.5. Anticancer effects

**Figure 6.**
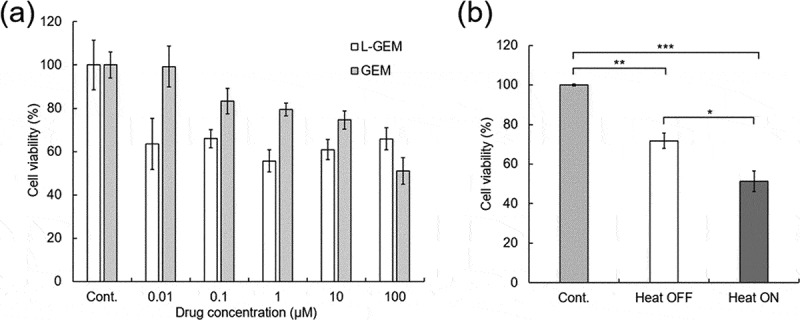
The cell viability of MIAPaCa-2 in (a) free GEM and L-GEM (n = 6) (b)different conditions of released drugs (* P ≤ 0.05, ** P ≤ 0.01, *** P ≤ 0.001, n = 5)

Since the released drug compound L-GEM has a maleimide chain (linker) attached onto it, which differs in structure to the native GEM, the cytotoxicity of L-GEM on MIAPaCa-II cells [32] was evaluated with different L-GEM concentrations (0.01–100 μM) for 24 h at 37°C. Anticancer effect of L-GEM was saturated at the concentration of 0.01 μM, and it was observed that approximately 34% of cells survived post treatment with 100 μM of L-GEM (Figure 6(a)). Figure 6(b) shows the survival numbers of cells after treatment with released L-GEM from the PFMA-L-GEM films. In this study, heating and cell culture experiments were conducted separately to prevent the effects of heating on the cells. First, the films were exposed to AMF for 60 min, and then the released L-GEM (supernatant solution) was collected and applied to MIAPaCa-II cells. For samples wherein heating was not conducted, approximately 28% of the cells were killed. These observations are consistent with the fact that approximately 38 μM of L-GEM was released from the film at 37 °C (Figure 5). On the contrary, approximately 49% of cells were killed, when L-GEM released from the film in the supernatant solution upon heating the cells. Although the number of cells surviving was not significantly decreased by heating, an enhanced cell killing effect was observed by thermally accelerated drug release based on the rDA reaction. Another advantage of using this system is that the structure of L-GEM can avoid deamination of cytidine in the DNA chain by activation-induced (cytidine) deaminase (AID), which enhances the bioavailability and the cytotoxicity effect [33].

## 4. Conclusions

This study demonstrates the efficiency of a thermally accelerated drug release system against pancreatic cancer cells using dynamic and reversible covalent chemistry. Anticancer prodrug GEM-tethered polymers (PFMA-L-GEM) were prepared with the DA reaction. The tethering bond was destructed by heating, resulting in a thermally accelerated drug release. By incorporating MNPs, the PFMA-L-GEM film could be heated by alternating magnetic field. *In vivo* experiments demonstrated that the film showed an enhanced cell-killing effect when pre-treated. These results proved that the temperature-triggered drug release platform might serve to preserve the payload at body temperature and can aid the rapid delivery of the drug within locally heated tissue to overcome side effects.
